# The Redcliffe Infirmary, Oxford

**Published:** 1893-04-29

**Authors:** 


### RADCLIFFE INFIRMARY, OXFORD.

We are glad to note that the citizens of Oxford are being aroused to a sense of the claims that this institution has upon the inhabitants of the city. The first of what we hope will prove many meetings to assist the building fund of the infirmary, was held at the mission rooms, St. Clement's, Oxford, when the vestry determined to institute a house-to-house collection, to organize an American sale, and generally to do their utmost to induce residents to contribute something, however small, to this fund. No doubt, if parochial action could be generally organized throughout Oxford, the £10,000 required will soon be collected. A committee was appointed, a house-to-house canvass agreed to, and it was resolved to ask the householders to contribute in cash or articles for the sale as they might prefer. The Edinburgh Royal Infirmary collects many hundreds a year by a house-to-house canvass of that city, and at Portsmouth the Royal Portsmouth Hospital, through Ward collections, on a similar plan, not only raises considerable sums in addition, but is enabled to continuously increase the annual subscription list. These two instances should be a great encouragement to the citizens of Oxford, and we hope that the movement initiated in the parish of St. Clement's will spread throughout the whole city. In such a case not only will the £10,000 be forthcoming for the building fund, but many hundreds a year will be added to the annual subscription list of the Radcliffe Infirmary.

